# Analysis of sleep duration, energy intakes, physical activity, and metabolic syndrome based on the presence or absence of obesity and hypertension in working Korean adults

**DOI:** 10.3389/fpubh.2025.1588706

**Published:** 2025-07-30

**Authors:** Eunsook Sung, Youngjun Lee, Sanghoo Kim, Jonghoon Park

**Affiliations:** ^1^Department of Exercise Rehabilitation, Sungshin Women’s University, Seoul, Republic of Korea; ^2^Department of Kinesiology, Michigan State University, East Lansing, MI, United States; ^3^Department of Physical Education, Korea University, Seoul, Republic of Korea

**Keywords:** hypertension, obesity, metabolic syndrome, sleep duration, physical activity, energy intake

## Abstract

**Background:**

Metabolic syndrome poses a serious public health concern among working adults, especially in the context of increasing rates of obesity and hypertension. These lifestyle-related conditions are intricately linked to sleep patterns, dietary behaviors, and physical activity.

**Objective:**

This study aimed to examine how sleep duration, energy intake, and physical activity levels—key lifestyle factors—are associated with metabolic syndrome based on the presence or absence of obesity and hypertension among working Korean adults.

**Methods:**

We analyzed 6,503 working Korean adults aged 20–59 years using cross-sectional data from the Korea National Health and Nutrition Examination Survey covering 2016–2018. This study divided the participants based on their obesity and hypertension status. We assessed the participants’ sleep duration on weekdays and weekends, their total dietary energy consumption and macronutrient intake using a 24-h dietary recall method conducted by trained interviewers, and occupational and recreational physical activities, and movement levels in MET min/week. We evaluated metabolic syndrome components using standard criteria and examined between-group differences using two-way analysis of variance and Bonferroni *post-hoc* tests.

**Results:**

Individuals with hypertension had significantly shorter weekday sleep (*p* < 0.05) and weekend sleep durations (*p* < 0.001) than those without. The highest total energy intake was significantly higher in obese individuals with hypertension than in those without hypertension (*p* < 0.05) and non-obese individuals with hypertension (*p* < 0.001). Furthermore, obese individuals with hypertension showed higher levels of occupational vigorous and total physical activities than those without hypertension (*p* < 0.01 and *p* < 0.001, respectively), whereas no difference was observed between obese and non-obese individuals with hypertension.

**Conclusion:**

This study concludes that hypertension and obesity may reinforce each other through reduced sleep duration, lower physical activity levels, and increased energy intake, thereby exacerbating metabolic syndrome. To prevent metabolic syndrome in working adults, multiple aspects of sleep, nutrition, and physical activity that match their unique risk profiles must be considered.

## 1 Introduction

The global public health landscape currently faces significant challenges such as obesity and hypertension. The World Health Organization has identified obesity as a major global health crisis and called it “globesity,” which now surpasses malnutrition in its contribution to illness and death rates (1). By 2030, obesity will affect almost one-fifth of women and one-seventh of men globally, resulting in more than 1 billion people being classified as obese (2). The complex nature of obesity emerges from behavioral patterns, including a decline in sleep quality, physical inactivity and unhealthy food choices (3–5). The prevalence of hypertension is increasing alongside obesity because both medical conditions frequently appear together and intensify their negative impact on cardiovascular well-being (6, 7). Hypertension is the primary risk factor for several cardiovascular diseases, including stroke and heart failure, making it a significant driver of morbidity and mortality rates worldwide (8–10).

Research has demonstrated that insufficient sleep causes people to consume more energy and adopt unhealthy eating habits while decreasing their physical activity, which leads to a higher risk of obesity and hypertension (11, 12). Sleep disturbances often result from obesity, leading to nocturnal symptoms such as obstructive sleep apnea and nocturia, which degrade sleep quality and contribute to a self-sustaining pattern of metabolic dysfunction (13, 14). Therefore, the relationship between obesity and sleep deprivation is highly complex and reciprocal, with each condition exacerbating the other through interrelated physiological and behavioral mechanisms. Moreover, individuals who consistently experience inadequate sleep show significant hormonal dysregulation, as evidenced by changes in leptin and ghrelin levels, which can lead to weight gain and vascular dysfunction (15, 16). These biological mechanisms underscore the importance of understanding lifestyle factors in working adults, which not only sustains the workforce but also represents a significant segment of the nation’s adult population.

These unique circumstances in Korean working adults and the pressures of the modern employment environment result in unpredictable sleep cycles (17), meal times (18), and physical inactivity (19), which increase the risk of metabolic syndrome (20). Working adults in South Korea face severe manifestations of these challenges. The distinctive occupational environment that features extended working hours and intense stress in addition to erratic timetables has led to general sleep deprivation (20, 21). According to national data, the prevalence of metabolic syndrome among Korean adults aged 20–59 years is estimated to be approximately 19% in men and 9% in women (22), which is lower than the overall adult prevalence of around 25–28% (23). This discrepancy reflects the relatively younger age of the working population but nonetheless underscores the urgent need for early lifestyle interventions. Early detection and intervention in this age group not only prevents long-term complications but also contributes to workforce sustainability and reduced healthcare burden at the population level.

According to the Korea National Health and Nutrition Examination Survey (KNHANES) 2016–2018 data, working adults in Korea with a higher body mass index (BMI) experience shorter sleep durations of <6 h per day, which may lead to metabolic problems and increased cardiovascular risk (24). This study highlights the complex bidirectional connections among obesity, high blood pressure, and lack of sleep, which call for specialized and comprehensive public health programs in the workplace. According to KNHANES VI (2013–2016) data analyses, people who follow traditional Korean balanced diets have lower rates of abdominal obesity, which is a primary risk factor for metabolic syndrome and hypertension (25, 26). Researchers examining individual dietary elements, including nut consumption and mineral intake, have shown that these foods may safeguard against high blood pressure (27). A study using KNHANES 2016–2018 data discovered that the prevalence of metabolic syndrome among young adults is strongly correlated with their physical activity levels and energy intake patterns, highlighting how lifestyle and nutritional elements combine to influence chronic disease conditions (28). These comprehensive findings indicate that detailed population-based surveillance reveals complex interactions among sleep quality, diet, physical activity, and cardiometabolic health in South Korea.

Research on lifestyle determinants affecting hypertension and obesity is important; however, research specifically focusing on working adults using nationally representative data remains limited. Representative large-scale studies serve as a vital foundation for the development of successful public health interventions. The KNHANES database provides detailed information that can be used to examine the associations among sleep duration, energy intake, physical activity, and metabolic health outcomes, specifically in individuals with obesity and hypertension. Therefore, by analyzing KNHANES data from 2016 to 2018, this study explicitly addresses this knowledge gap by examining how modifiable lifestyle factors interact with metabolic syndrome and obesity to influence hypertension levels in working Korean adults. We aimed to develop focused strategies to reduce the effects of these conditions and their associated metabolic disorders.

## 2 Materials and methods

### 2.1 Sample and design

This study used cross-sectional data from the Korea National Health and Nutrition Examination Survey (KNHANES) conducted by the Korea Centers for Disease Control and Prevention (KCDC) from 2016 to 2018. These data are updated every 3 years. Therefore, we used current data. The details of the study design and data source profiles followed the methods outlined in the guidelines for using raw KNHANES data and in the final report on the sampling frame (29).

**Figure 1:**
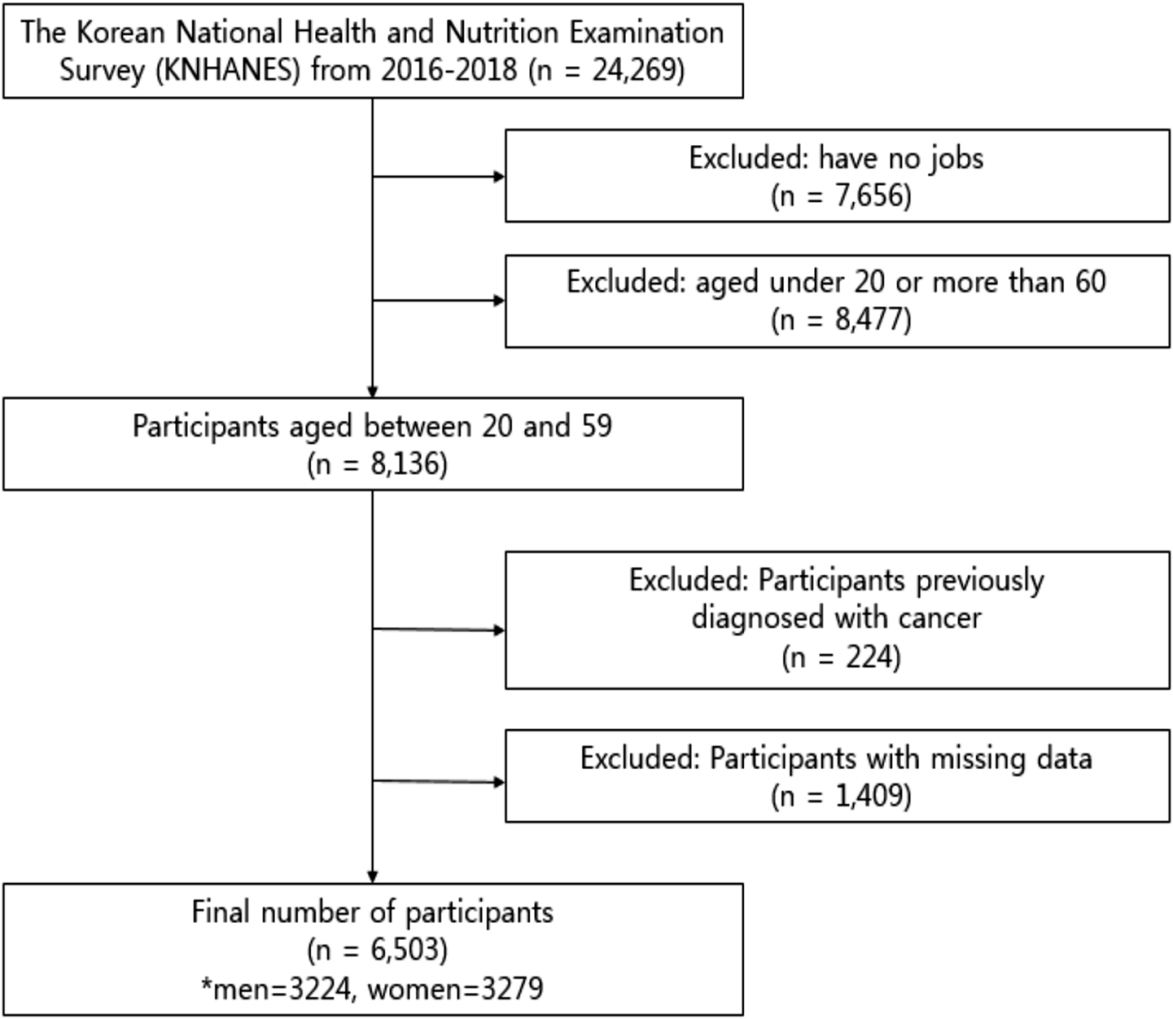
Flow diagram for the selection of study participants.

From 2016 to 2018, 24,269 individuals completed a health interview survey, nutrition surveys, and health examinations, which were conducted according to the Declaration of Helsinki. This is a survey to assess the health and nutritional status of South Koreans, and it was conducted by the Korea Centers for Disease Control and Prevention. The National Health and Nutrition Examination Survey was approved by the Institutional Review Board of the Korea Centers for Disease Control and Prevention (reference number 2018-01-03-P-A). Preceding the survey, all participants were informed about the purpose and procedures of the survey, and written informed consent was obtained from each participant prior to their involvement in the survey. 7,656 participants without jobs were excluded, and 8,477 participants under the age of 20 and over 60 years were excluded. Among the 8,136 participants aged 20 to 59 years, 224 individuals who had been previously diagnosed with or treated for cancer (stomach cancer, colorectal cancer, liver cancer, cervical cancer, breast cancer, thyroid cancer, lung cancer, and other cancers) and 1,409 participants with missing data were excluded from the study (Figure 1). The final sample size of the study comprised 6,503 (men, 3,224; women, 3,279) working adult males ranging in age from 20 to 50 years and on the day of the survey, the participants were not taking medication for chronic diseases (e.g., hypertension, hyperlipidemia, diabetes).

### 2.2 Measures

We evaluated the components of Metabolic syndrome by determining waist circumference alongside blood pressure, fasting blood glucose levels, triglyceride (TG) concentrations, and high-density lipoprotein cholesterol (HDL-C) levels. The survey data from KNHANES and interview responses provided the measurements and variables related to sleep duration, physical activity, and energy intake. The GPAQ served as the measurement tool for physical activity, while dietary intake data was gathered through 24-h recall interviews.

### 2.3 Metabolic syndrome components

The diagnosis of metabolic syndrome was made in accordance with the National Cholesterol Education Program-Adult Treatment Panel III (30) and the new combined guidelines of the American Heart Association and the National Heart, Lung, and Blood Institute (31). For waist circumference, we used the criteria proposed by the Korean Society for Obesity (32). Metabolic syndrome was diagnosed if participants had three or more of the following (Huang, 2009): waist circumference >90 cm (male) or >80 cm (female), systolic blood pressure (SBP) ≥ 130 mmHg or diastolic blood pressure (DBP) ≥ 85 mmHg, fasting TG levels ≥150 mg/dL, fasting HDL-C levels <40 mg/dL (male) or <50 mg/dL (female), and fasting glucose (FG) levels ≥110 mg/dL.

### 2.4 Sleep characteristics

In this study, participants completed a self-administered questionnaire as part of the KNHANES survey to assess sleep duration. Through separate responses, participants indicated their typical bedtime and wake-up time for weekdays and weekends. The researchers computed the daily average sleep duration expressed in minutes from this data. Previous research with the KNHANES dataset has validated this method (33). No composite score or sleep quality category was used, as the survey did not include validated sleep quality items.

### 2.5 Physical activity

The GPAQ comprises 16 questions grouped to capture PA in different behavioral domains: work, transport, and recreational activities. Five domains of PA were analyzed: vigorous-intensity work, moderate-intensity work, transport, vigorous-intensity recreation, and moderate-intensity recreation. Participants answered the five domains freely, without any additional options regarding how many times a week and how many minutes per day they performed the activity. The WHO GPAQ analysis guidelines were used to analyze the GPAQ data (34). We estimated that a person’s caloric expenditure was four times higher when they were moderately active and eight times higher when they were vigorously active compared to sitting quietly. Therefore, when calculating the total energy expenditure of an individual using GPAQ data, four METs were assigned to the time spent in moderate activity. Eight METs were assigned to the time spent in vigorous activity, and the details are as follows:

- Vigorous intensity activity: occupational (MET) = 8.0 × vigorous intensity physical activity (day/week) × 1-day vigorous intensity physical activity (minutes/day)
- Moderate intensity activity: occupational (MET) = 4.0 × moderate intensity physical activity (day/week) × 1-day moderate intensity physical activity (minutes/day)
- Vigorous intensity activity: recreational (MET) = 8.0 × vigorous intensity physical activity (day/week) × 1-day vigorous intensity physical activity (minutes/day)
- Moderate intensity activity: recreational (MET) = 4.0 × moderate intensity physical activity (day/week) × 1-day moderate intensity physical activity (minutes/day)
- Place movement (MET) = 4.0 × place movement physical activity (day/week) × 1-day place movement physical activity
- Total Physical Activity (MET) = vigorous intensity activity: occupational + moderate intensity activity: occupational + vigorous intensity activity: recreational + moderate intensity activity: recreational + place movement.

PA levels were divided into four groups: inactive (0–249 MET min/week), somewhat active (250–499 MET min/week), active (500–999 MET min/week), and very active (>1,000 MET min/week). These thresholds are based on their equivalence to the following PA thresholds: 250 MET min/week corresponds to an energy expenditure dose equal to half the threshold, 500 MET min/week corresponds to the minimum threshold, and 1,000 MET min/week corresponds to twice the minimum threshold (35).

### 2.6 Energy intake

Dietary outcomes were obtained using the 24-h recall method by interviewing target households in person. Nutrition survey data were collected from participants’ homes by trained dietitians 1 week after the health interview and health examination. The daily energy intake was calculated using the Korean Food and Nutrient Database of the Rural Development Authority. The following items were included in the analyses: total energy intake, carbohydrate intake, protein intake, and daily fat intake. Energy intake data were converted into kcal using the conversion factor of 4 kcal/g for carbohydrates and proteins and 9 kcal/g for fats (36).

### 2.7 Statistical analysis

Continuous variables were presented as means and standard errors. Normality of the distribution of all outcome-variable data was verified using the Kolmogorov–Smirnov test. An independent t-test was used to analyze risk factors for MetS, as well as sleep duration, energy intakes and PA levels between the hypertension (HT) and non-HT groups. Two-way analyses of variance (ANOVA) were used to analyze the differences in risk factors for MetS, as well as sleep duration, energy intakes and PA levels between participants with and without HT, and between obese and non-obese participants. Partial eta-squared (η^2^) values were calculated to represent effect sizes. If a significant interaction effect was found by two-way ANOVA, a Bonferroni *post-hoc* test was used to perform multiple pairwise comparisons while controlling for the familywise error rate. This correction was consistently applied to all relevant group comparisons. It compares the obese-specificity of dependent variables in each group (with and without HT) separately. Statistical analyses were performed using SPSS version 25.0 for Windows (IBM Corp., Armonk, NY, United States). The level of significance was set at 0.05.

## 3 Results

A total of 24,269 individuals completed health interviews, nutrition surveys, and examinations. After excluding 7,656 individuals without employment and 8,477 individuals aged below 20 or above 59 years, 8,136 working-age adults remained. We further excluded 224 individuals with a cancer diagnosis and 1,409 with missing data, resulting in a final analytical sample of 6,503 participants (men: 3,224; women: 3,279). The flow of participant selection is presented in Figure 1.

Table 1 presents the characteristics of 6,503 working adults, including 2,250 individuals with obesity (34.6%) and 870 individuals with hypertension (13.4%). The KNHANES health examination survey evaluated essential metabolic syndrome components, including waist circumference, blood pressure, fasting glucose, triglycerides, and HDL-C levels. Our study collected sleep duration data through self-administered questionnaires and physical activity data from the Global Physical Activity Questionnaire (GPAQ), which was reported in MET minutes/week. The dietary energy intake alongside macronutrient consumption was assessed through 24-h recall interviews administered by trained staff. Participants with hypertension were significantly older than their those without hypertension overall and those in the obese and non-obese groups (*p* < 0.001). These participants were taller (169.1 ± 0.3 cm vs. 166.9 ± 0.1 cm, *p* < 0.001), heavier (75.2 ± 0.5 kg vs. 66.3 ± 0.2 kg, *p* < 0.001), and had higher BMIs across all groups (*p* < 0.001). Individuals with hypertension reported higher levels of alcohol consumption overall (*p* < 0.001) and those in the non-obese (*p* < 0.001) and obese (*p* = 0.027) groups. Smoking prevalence was higher among individuals with hypertension overall (*p* < 0.001) and those in the non-obese group (*p* < 0.001); however, no significant difference was observed among participants in the obese group.

**Table 1:** Descriptive characteristics of the participants.

Variables	Total (*n* = 6,503)			Non-obese (*n* = 4,253)			Obese (*n* = 2,250)		
	Non-HT (*n* = 5,633)	HT (*n* = 870)	*p*-value	Non-HT (*n* = 3,894)	HT (*n* = 359)	*p*-value	Non-HT (*n* = 1,739)	HT (*n* = 511)	*p*-value
Age (years)	40.6 ± 0.2	45.5 ± 0.4	**<0.001** ^***^	40.2 ± 0.2	47.3 ± 0.7	**<0.001** ^***^	41.3 ± 0.4	44.3 ± 0.4	**<0.001** ^***^
Height (cm)	166.9 ± 0.1	169.1 ± 0.3	**<0.001** ^***^	166.1 ± 0.2	167.5 ± 0.5	**0.007** ^**^	168.5 ± 0.2	170.2 ± 0.4	**<0.001** ^***^
Body weight (kg)	66.3 ± 0.2	75.2 ± 0.5	**<0.001** ^***^	60.5 ± 0.2	63.9 ± 0.5	**<0.001** ^***^	78.6 ± 0.3	83.0 ± 0.6	**<0.001** ^***^
BMI $\left(\mathrm{kg}/m^{2}\right)$	23.7 ± 0.1	26.2 ± 0.1	**<0.001** ^***^	21.8 ± 0.0	22.7 ± 0.1	**<0.001** ^***^	27.6 ± 0.1	28.6 ± 0.1	**<0.001** ^***^
Alcohol	67.1	75.6	**<0.001** ^***^	65.5	75.4	**<0.001** ^***^	70.6	75.8	**0.027** ^*^
Smoking	25.2	32.3	**<0.001** ^***^	22.2	34.1	**<0.001** ^***^	31.5	31.1	0.897

Values are expressed as means ± standard errors. BMI, body mass index; HT, hypertension. *p* < 0.05, *p* < 0.01, **p < 0.001 compared between non-HT and HT groups within each column group (Total, Non-Obese, Obese). Bold values indicate statistically significant differences. Statistical differences were assessed using independent t-tests for continuous variables and chi-square tests for categorical variables.

The differences in metabolic syndrome components, according to the presence or absence of hypertension and obesity are presented in Table 2. In the entire study population, individuals with hypertension had a significantly higher waist circumference, triglyceride (TG) levels, systolic blood pressure (SBP), diastolic blood pressure (DBP), and fasting glucose levels and significantly lower high-density lipoprotein cholesterol (HDL-C) levels than those without hypertension (all *p* < 0.001). The interaction between hypertension status and obesity was statistically significant for waist circumference; TG, HDL-C, and fasting glucose levels; and DBP (all *p* < 0.001). Bonferroni *post-hoc* tests revealed that, among non-obese participants, those with hypertension had a significantly larger waist circumference, higher TG and fasting glucose levels, and higher SBP and DBP (all *p* < 0.001) but lower HDL-C levels (*p* < 0.001) than those without. Similarly, obese participants with hypertension showed a significantly larger waist circumference, higher TG and fasting glucose levels, and higher SBP and DBP (all *p* < 0.001) than those without hypertension. Furthermore, individuals with obesity and hypertension had a significantly greater waist circumference, TG levels (*p* < 0.001), and DBP (*p* < 0.01) but lower HDL-C levels (*p* < 0.001) than those without obesity and hypertension.

**Table 2:** Metabolic syndrome components.

Variables	Group	Total	Obesity		ANOVA			
			Non-obese	Obese	F-value		*p*-value $\left(\eta^{2}\right)$	Power
Waist circumference (cm)	Non-HT HT *p*-value	81.0 ± 0.2 89.0 ± 0.4 <0.001***	76.2 ± 0.1 81.0 ± 0.4^**†††**^	90.9 ± 0.2^**###**^ 94.5 ± 0.4^**###, †††**^	O H O × H	977.462 409.987 17.352***	0.000(0.088) 0.000(0.020) 0.000(0.002)	1.000 1.000 1.000
TG (mg/dL)	Non-HT HT *p*-value	131.8 ± 2.0 205.0 ± 6.4 <0.001***	115.2 ± 2.0 186.8 ± 10.4^**†††**^	167.1 ± 3.6^**###**^ 217.6 ± 7.9^**###, †††**^	O H O × H	114.953 394.885 6.682**	0.000 (0.012) 0.000 (0.020) 0.001 (0.001)	1.000 1.000 0.916
HDL-C (mg/dL)	Non-HT HT *p*-value	51.8 ± 0.2 48.5 ± 0.4 <0.001***	54.2 ± 0.3 51.6 ± 0.7^**†††**^	46.7 ± 0.3^**###**^ 46.4 ± 0.5^**###**^	O H O × H	118.739 28.657 4.364*	0.000 (0.012) 0.000 (0.001) 0.013 (0.000)	1.000 1.000 0.757
SBP (mmHg)	Non-HT HT *p*-value	111.6 ± 0.2 138.5 ± 0.5 <0.001***	109.6 ± 0.2 139.4 ± 0.8^**†††**^	115.8 ± 0.3^**###**^ 137.8 ± 0.7^**†††**^	O H O × H	137.595 9519.607 145.531***	0.000 (0.013) 0.000 (0.319) 0.000 (0.014)	1.000 1.000 1.000
DBP (mmHg)	Non-HT HT *p*-value	74.6 ± 0.1 94.4 ± 0.3 <0.001***	73.2 ± 0.1 93.2 ± 0.4^**†††**^	77.6 ± 0.2^**###**^ 95.3 ± 0.4^**##, †††**^	O H O × H	626.477 6622.656 83.088***	0.000 (0.058) 0.000 (0.246) 0.000 (0.008)	1.000 1.000 1.000
Fasting glucose (mg/dL)	Non-HT HT *p*-value	96.9 ± 0.4 106.7 ± 1.0 <0.001***	94.5 ± 0.3 104.7 ± 1.9^**†††**^	101.7 ± 0.7^**###**^ 108.0 ± 1.4^**†††**^	O H O × H	24.052 234.090 3.744*	0.000 (0.002) 0.000 (0.012) 0.024 (0.000)	1.000 1.000 0.687

Values are expressed as means standard errors; HT, hypertension; TG, triglyceride; HDL-C, high-density lipoprotein cholesterol; SBP, systolic blood pressure; DBP, diastolic blood pressure; HbA1c, glycated hemoglobin. *p < 0.05, **p < 0.01, ***p < 0.001 compared between non-HT and HT groups; ^†††^*p* < 0.001 compared with Non-HT values, ^#^*p* < 0.05, ^##^*p* < 0.01, ^###^*p* < 0.001 compared with Non-Obese values. Two-way analysis of variance following Bonferroni *post-hoc* test. Main effect = H (Hypertension) and O (Obesity), Interaction effect = H × O (Hypertension × Obesity), O × H is statistically significant if the *p* value is less than 0.05 and has been indicated in bold in the table.

Differences in sleep characteristics according to the presence or absence of hypertension and obesity are presented in Table 3. In the entire population, individuals with hypertension slept significantly less frequently on weekdays than those without (*p* < 0.05). However, there was no significant difference in weekday sleep duration between individuals with and without hypertension in each obesity subgroup. Individuals with hypertension also had significantly shorter sleep durations on weekends than those without (*p* < 0.001). The interaction between hypertension status and obesity was statistically significant for weekend sleep duration (*p* = 0.013). Bonferroni *post-hoc* tests revealed that individuals with obesity and hypertension had a significantly shorter weekend sleep duration than those without obesity and hypertension (*p* < 0.05) and those with obesity and without HT (*p* < 0.01).

**Table 3:** Sleep characteristics.

Variables	Group	Total	Obesity		ANOVA			
			Non-Obese	Obese	F-value		*p*-value $\left(\eta^{2}\right)$	Power
Average sleep time per day on weekday (minute)	Non-HT HT *p*-value	417.8 ± 1.3 411.0 ± 2.5 0.010^*^	420.7 ± 1.4 413.7 ± 4.6	411.7 ± 2.4 409.2 ± 2.8	O H O × H	17.026 2.012 1.552	0.000 (0.002) 0.156 (0.000) 0.212 (0.000)	1.000 0.294 0.331
Average sleep time per day on weekend (minute)	Non-HT HT *p*-value	472.3 ± 1.3 456.2 ± 3.3 <0.001^***^	477.3 ± 1.6 466.2 ± 5.2^**†**^	461.5 ± 2.2^**###**^ 449.3 ± 4.2^**#, ††**^	O H O × H	15.340 45.205 4.374^*^	0.000 (0.002) 0.000 (0.002) 0.013 (0.000)	0.999 1.000 0.758

Values are expressed as means standard errors; HT, hypertension. *p < 0.05, ***p < 0.001 compared between non-HT and HT groups; ^†^*p* < 0.05, ^††^*p* < 0.01 compared with Non-HT values, ^#^*p* < 0.05, ^###^*p* < 0.001 compared with Non-Obese values. Two-way analysis of variance following Bonferroni *post-hoc* test. Main effect = H (Hypertension) and O (Obesity), Interaction effect = H × O (Hypertension × Obesity), O × H is statistically significant if the *p* value is less than 0.05 and has been indicated in bold in the table.

Differences in energy intake according to the presence or absence of hypertension and obesity are presented in Table 4. In the entire population, individuals with hypertension had a significantly higher total energy intake than those without (*p* < 0.001). The interaction between hypertension status and obesity was statistically significant for total energy intake (*p* < 0.001). Bonferroni *post-hoc* tests revealed that individuals with obesity and hypertension had the highest total energy intake, which was significantly greater than that of those without obesity and hypertension (*p* < 0.05) and those without obesity and with HT (*p* < 0.001). A significant interaction effect was observed for carbohydrate intake (*p* = 0.003). Bonferroni *post-hoc* tests indicated that obese individuals consumed significantly more carbohydrates than non-obese individuals (*p* < 0.001). Among obese participants, those with hypertension had significantly higher carbohydrate intake than those without (*p* < 0.05). However, no significant difference in carbohydrate intake was found between individuals with and without hypertension in the non-obese group. Protein intake exhibited a statistically significant interaction effect (*p* < 0.001). Bonferroni *post-hoc* tests showed that obese individuals had a significantly higher protein intake than non-obese individuals (*p* < 0.001); however, there was no significant difference in protein intake between individuals with and without hypertension in either the non-obese or obese group. A significant interaction effect was observed for fat intake (*p* < 0.001). Bonferroni *post-hoc* tests revealed that obese individuals consumed significantly more fat than non-obese individuals (*p* < 0.001). In the non-obese group, individuals with hypertension had a significantly lower fat intake than those without (*p* < 0.01). However, no significant difference in fat intake was observed between individuals with and without hypertension in the obese group.

**Table 4:** Energy intakes.

Variables	Group	Total	Obesity		ANOVA			
			Non-Obese	Obese	F-value		*p*-value $\left(\eta^{2}\right)$	Power
Total energy intake (kcal/day)	Non-HT HT *p*-value	2189.7 ± 15.7 2334.5 ± 37.7 <0.001***	2129.2 ± 16.9 2203.5 ± 56.0	2318.7 ± 31.0^###^ 2422.4 ± 50.8^###, **†**^	O H O × H	262.856 0.001 21.499^***^	0.000 (0.029) 0.982 (0.000) 0.000 (0.002)	1.000 0.050 1.000
Carbohydrate intake (kcal/day)	Non-HT HT *p*-value	1230.8 ± 9.5 1264.1 ± 19.4 0.110	1213.1 ± 10.2 1189.7 ± 28.4	1268.5 ± 16.2^###^ 1314.0 ± 27.1^###, **†**^	O H O × H	47.150 0.479 5.824^**^	0.000 (0.005) 0.489 (0.000) 0.003 (0.001)	1.000 0.106 0.873
Protein intake (kcal/day)	Non-HT HT *p*-value	323.1 ± 3.0 327.8 ± 6.8 0.508	310.0 ± 3.0 301.8 ± 10.1	351.0 ± 5.6^###^ 345.2 ± 8.6^###^	O H O × H	238.459 20.615 10.614^***^	0.000 (0.027) 0.000 (0.001) 0.000 (0.001)	1.000 0.995 0.990
Fat intake (kcal/day)	Non-HT HT *p*-value	486.9 ± 5.9 465.3 ± 12.1 0.080	471.7 ± 7.8 419.4 ± 17.2^**††**^	519.3 ± 10.4^###^ 496.1 ± 16.6^###^	O H O × H	212.125 67.756 17.755^***^	0.000 (0.024) 0.000 (0.004) 0.000 (0.002)	1.000 1.000 1.000

Values are expressed as means standard errors; HT, hypertension. ***p < 0.001 compared between non-HT and HT groups; ^†^*p* < 0.05, ^††^*p* < 0.01 compared with Non-HT values, ^###^*p* < 0.001 compared with Non-Obese values. Two-way analysis of variance following Bonferroni *post-hoc* test. Main effect = H (Hypertension) and O (Obesity), Interaction effect = H × O (Hypertension × Obesity), O × H is statistically significant if the *p* value is less than 0.05 and has been indicated in bold in the table.

Differences in physical activity levels according to the presence or absence of hypertension and obesity are presented in Table 5. A significant interaction effect was observed for vigorous occupational activity (*p* = 0.001). Bonferroni *post-hoc* tests revealed that individuals with obesity and without hypertension engaged in significantly more vigorous occupational activities than those without obesity (*p* < 0.001). Among obese participants, those with hypertension demonstrated significantly lower levels of vigorous occupational activity than those without (*p* < 0.01). Total physical activity also showed a significant interaction effect (*p* = 0.002). Bonferroni *post-hoc* tests indicated that individuals with obesity and without hypertension participated in significantly more total physical activity than those without obesity and hypertension (*p* < 0.001). Additionally, among participants with obesity, individuals with hypertension exhibited significantly lower total physical activity levels than those without (*p* < 0.001).

**Table 5:** Levels of physical activity.

Physical activity (MET min/week)	Group	Total	Obesity		ANOVA			
			Non-Obese	Obese	F-value		*p*-value $\left(\eta^{2}\right)$	Power
Occupational vigorous	Non-HT HT *p*-value	97.4 ± 15.9 81.0 ± 34.3 0.667	65.9 ± 15.7 128.2 ± 77.3	164.2 ± 34.0^###^ 48.3 ± 20.5^**††**^	O H O × H	3.632 0.223 7.297**	0.026 (0.000) 0.637 (0.000) 0.001 (0.001)	0.673 0.076 0.938
Occupational moderate	Non-HT HT *p*-value	197.0 ± 21.4 208.3 ± 43.4 0.812	153.5 ± 21.0 200.9 ± 73.1	288.9 ± 29.5 213.3 ± 52.7	O H O × H	41.703 0.995 2.785	0.000 (0.004) 0.319 (0.000) 0.062 (0.000)	1.000 0.169 0.551
Place movement	Non-HT HT *p*-value	454.3 ± 17.1 430.0 ± 29.9 0.460	446.4 ± 17.4 434.0 ± 42.8	470.8 ± 26.6 427.2 ± 37.0	O H O × H	2.012 0.350 1.666	0.134 (0.000) 0.554 (0.000) 0.189 (0.000)	0.418 0.091 0.353
Recreational vigorous	Non-HT HT *p*-value	176.5 ± 10.7 164.8 ± 25.6 0.676	158.7 ± 12.2 141.8 ± 39.3	214.3 ± 19.4 180.7 ± 31.8	O H O × H	19.244 4.621 0.234	0.000 (0.002) 0.032 (0.000) 0.791 (0.000)	1.000 0.575 0.087
Recreational moderate	Non-HT HT *p*-value	164.0 ± 6.5 161.0 ± 13.2 0.835	154.3 ± 7.7 166.7 ± 22.8	184.6 ± 10.8 157.0 ± 16.8	O H O × H	10.916 1.834 1.450	0.000 (0.001) 0.176 (0.000) 0.235 (0.000)	0.991 0.273 0.312
Total physical activity	Non-HT HT *p*-value	1089.2 ± 44.6 1045.0 ± 69.5 0.588	978.8 ± 44.0 1071.7 ± 131.5	1322.8 ± 78.1^###^ 1026.5 ± 80.2^**†††**^	O H O × H	42.123 3.704 6.007**	0.000 (0.004) 0.054 (0.000) 0.002 (0.001)	1.000 0.486 0.883

Values are expressed as means standard errors; HT, hypertension. ^††^*p* < 0.01, ^†††^*p* < 0.001 compared with Non-HT values, ^###^*p* < 0.001 compared with Non-Obese values. Two-way analysis of variance following Bonferroni *post-hoc* test. Main effect = H (Hypertension) and O (Obesity), Interaction effect = H × O (Hypertension × Obesity), O × H is statistically significant if the *p* value is less than 0.05 and has been indicated in bold in the table.

## 4 Discussion

In this study, we analyzed how sleep duration, energy intake, physical activity levels, and metabolic syndrome components vary among working Korean adults with hypertension and obesity. Individuals with both obesity and hypertension demonstrated severe effects of metabolic syndrome–related prevalence factors. Our data demonstrated that people with hypertension experienced shorter average sleep durations on weekdays and weekends, and that weekend sleep duration significantly decreased in patients with both obesity and hypertension. People with hypertension or obesity consume more total energy daily than healthy individuals. Furthermore, people with both obesity and hypertension consume more calories throughout the day than individuals with either condition alone. Occupational vigorous activity and total physical activity levels were higher among obese individuals without hypertension; people with obesity and hypertension demonstrated significantly reduced levels of these activities compared with those in other groups. This study demonstrated how hypertension and obesity significantly affect sleep duration, energy intake, and physical activity levels in working Korean adults, which likely aggravates the prevalence of metabolic syndrome.

In this study, the obesity and hypertension rates among Korean working adults were 34.6 and 13.4%, respectively (Table 1). A previous study using KNHANES data from 2019 to 2021 reported that the obesity rate in Korean adults ranges between 30 and 35%, which is in good agreement with our findings (37). However, according to our data, working adults experience lower hypertension rates (13.4%) than the general adult population (~25–28%) (23). This discrepancy is likely due to the younger age distribution in the working population, as also supported by our finding that hypertensive participants were significantly older than their non-hypertensive counterparts (*p* < 0.001). However, the lower observed hypertension may also be partially influenced by self-report limitations or undiagnosed cases, which are common in national health surveys using interview-based data.

This study established that individuals with both obesity and hypertension had all metabolic syndrome–related disease factors, with the poorest outcomes observed in these patients (Table 2). Obesity is a key element of metabolic syndrome and is strongly associated with hypertension, diabetes, and dyslipidemia (38). Visceral fat accumulation contributes to insulin resistance, inflammation, and oxidative stress and accelerates the development of metabolic syndrome (39, 40). Additionally, obesity impairs kidney function, leading to hypertension and further worsening of metabolic syndrome (41). Hypertension plays a critical role in metabolic syndrome and contributes to its development and progression, along with obesity. Hypertension exacerbates these conditions through endothelial dysfunction and oxidative stress (42, 43). The combination of obesity and hypertension results in more severe interactions, leading to heightened health risks (44, 45). The KNHANES 2019 data confirmed that this combination elevates metabolic risk markers, including dyslipidemia and fasting glucose levels (46). Further studies, such as that on 2010–2012 KNHANES data, have shown that the resting heart rate in individuals with obesity and hypertension is correlated with insulin resistance and metabolic syndrome components (47). Moreover, the 2019–2020 KNHANES data demonstrated that early obesity increases the risk of metabolic syndrome in adulthood (48). While the mechanisms linking obesity and hypertension to metabolic syndrome are well-established, previous studies have often focused on either older populations or disease-specific samples. Our findings add to the literature by confirming these associations in a working-age cohort, where early intervention may be more feasible and impactful. However, it is important to note that our cross-sectional design limits causal inference, and the possibility of residual confounding (e.g., undetected comorbidities or medication use) should be considered when interpreting these relationships.

Our findings showed that individuals with hypertension experienced reduced sleep time on weekdays and weekends, with those with obesity and hypertension experiencing even shorter weekend sleep durations (Table 3). This aligns with that reported in previous studies linking short sleep duration to an increased risk of obesity and hypertension (11, 12). Studies have confirmed that individuals sleeping fewer than 6 h per night are more prone to both conditions (49). Short sleep duration leads to metabolic dysregulation, increased appetite, and reduced physical activity, thereby contributing to obesity and hypertension. Furthermore, insufficient sleep is associated with higher energy intake and lower energy expenditure, further exacerbating these risks (50). Our findings are particularly meaningful as they focus on a working-age Korean population—a group likely to be vulnerable to sleep disturbances due to irregular schedules, long work hours, and job-related stress. This occupational context adds a unique perspective to the growing body of evidence linking short sleep to metabolic dysfunction.

For instance, Baek et al. (51) and Son et al. (52) introduced the concept of “Sunday night insomnia,” wherein work-related anxiety before the Monday workweek significantly shortens weekend sleep—an effect we also observed in the comorbid obesity and hypertension group (51, 52). An analysis of 41,805 Korean adults from KNHANES 2007–2015 revealed that adults sleeping 5 h or less per night had significantly higher risks of both general and abdominal obesity, with particularly strong associations in those aged 30–49 years (53). Moreover, KNHANES 2001–2005 data highlighted that insufficient sleep increases metabolic dysfunction, raising the likelihood of obesity and hypertension (54). However, as with other studies using large national survey data, our measurement of sleep was based on self-reported bedtime and wake-up time, which may be subject to recall bias or misclassification. Despite this limitation, the associations we observed support the growing evidence that short sleep duration plays a critical role in the development and progression of cardiometabolic disorders. Improving sleep hygiene and protecting weekend recovery sleep may represent an effective intervention point for metabolic health, particularly among working adults.

People with hypertension consumed significantly more total daily energy, whereas those with obesity showed a higher intake of all macronutrient components (Table 4). The total daily energy intake was notably higher among those with both obesity and hypertension. This aligns with that reported in the literature showing that excessive energy intake increases the risk of both obesity and hypertension as it leads to weight gain and an elevated hypertension risk (55). Individuals with obesity also consume higher amounts of calories in the form of carbohydrates, fats, and proteins, which contributes to poor dietary quality and related metabolic disorders (56). Studies using NHANES and KNHANES 2007–2012 data revealed that high carbohydrate consumption causes metabolic issues in adults in the United States (US) and South Korea, with stronger impacts observed in Koreans. This may be attributed to the Korean diet, where approximately 66% of daily energy is derived from carbohydrates, compared with 50% in the US diet. Such diets have been linked to lower HDL cholesterol levels and higher triglyceride levels (57). Our findings extend this evidence by showing that Korean working adults with both obesity and hypertension have the highest daily energy intake, suggesting a compounding effect of these two conditions on dietary behavior. The traditional high-carbohydrate Korean diet, when combined with excessive energy consumption, worsens metabolic risk factors. Additionally, sugar-sweetened beverage consumption is an independent risk factor for obesity and metabolic syndrome in Koreans. Women who consume these beverages daily have significantly higher odds of developing these conditions than non-consumers (58). While these findings align with prior research, it is important to acknowledge that our dietary data were based on a single 24-h recall, which may be subject to underreporting or overreporting, particularly in overweight individuals (59). The management of metabolic syndrome requires strict energy intake control, particularly in patients with both conditions. Previous findings indicated that prohibiting the consumption of sugary snacks and beverages significantly improves body composition (60, 61). As sugar consumption drives excessive energy intake and contributes to obesity and hypertension (62, 63), integrating sugar restrictions into comprehensive management plans is crucial for reducing complications and enhancing metabolic health. Future interventions for this population should not only target total caloric intake, but also emphasize the cultural context of carbohydrate-dominant diets and high sugar beverage consumption, which may have a disproportionate metabolic impact on Korean adults.

Our study demonstrated that individuals with obesity but without hypertension engaged in greater vigorous occupational and total physical activities (Table 5). Although occupational category data were not collected in our study, this pattern may reflect higher physical demands in blue-collar roles, a trend also observed in previous Korean studies. These individuals may also participate in physical activities as a compensatory mechanism to manage their weight and prevent hypertension. Previous research supports these findings, showing that individuals with obesity who engage in more physical activity have a reduced risk of developing hypertension (64). By contrast, individuals with obesity and hypertension exhibited significantly lower levels of vigorous occupational and total physical activities. Hypertension tends to reduce physical activity levels owing to fatigue, decreased physical fitness, and heightened cardiovascular strain (65, 66). Moreover, the combination of obesity and hypertension exacerbates these challenges, leading to a further reduction in physical activity (67). These findings are consistent with a large body of literature suggesting that hypertension is both a cause and consequence of physical inactivity. Our study adds to this by showing how this pattern manifests specifically within a working-age Korean population, a group underrepresented in international physical activity research. KNHANES 2016–2017 data on middle-aged and older women showed that frequent occupational physical activity combined with extended sedentary time increased the risk of hypertension, even among those following aerobic exercise routines (68). However, the self-reported nature of physical activity data in KNHANES should be acknowledged as a limitation, as overestimation of activity is common among adults with overweight or chronic conditions (69). These results underscore the importance of targeted interventions that promote not only physical activity but also recovery, ergonomics, and flexible exercise strategies suited to the capabilities of individuals with obesity and hypertension. In particular, integrated programs that also address sleep quality may enhance intervention adherence and long-term impact (4, 60, 61, 70).

This study has several limitations. First, our evaluation of working adults with metabolic syndrome did not account for the timing of the first appearance or how long it lasted, limiting our understanding of how the progression or chronicity of the condition might influence the outcomes. Second, the evaluation of sleep duration or physical activity levels was conducted using survey data instead of objective measurements, such as heart rate monitoring or accelerometers, which might produce inaccurate results. This reliance on self-reporting introduces the possibility of recall bias and may lead to discrepancies between the reported and actual behaviors. Prior studies have shown that recall bias can lead to an overestimation or underestimation of physical activity levels by up to 35%, and similar biases have been reported in sleep and dietary self-reports (59, 69). Nevertheless, since all participants were assessed using the same self-report instruments under standardized procedures, any recall bias is likely to be non-differential and would bias results toward the null. Furthermore, the large, nationally representative sample and use of validated survey methods in KNHANES support the overall robustness and generalizability of the findings. Third, this study found simple differences in sleep duration, energy intake, and physical activity without proving any causal links. Fourth, the 24-h recall approach for dietary evaluation may not accurately capture long-term eating habits because it depends on participants’ reports of their food and beverage consumption from the previous day. Our analysis revealed 467 low-reporting females, 47 over-reporting females, 264 low-reporting males, and 62 over-reporting males. Despite these limitations, this study demonstrates its strengths through a detailed assessment of multiple lifestyle factors, including sleep duration, energy intake, and physical activity levels, in Korean working adults with hypertension and/or obesity. This is particularly notable because most previous studies on the relationship among metabolic syndrome, physical activity, and energy intake have primarily focused on older adults.

Nevertheless, this study has several notable strengths. Firstly, this study initially used information from the KNHANES, which represents the national population and includes a substantial sample size to ensure that findings can apply to Korea’s general working adult population. Second, this study differed from earlier research that looked at obesity or hypertension separately by examining how both conditions interacted through multiple health aspects such as sleep patterns, physical activity levels, and energy consumption. Third, the study reveals rare behavioral and physiological features since it examines a working-age population, which metabolic health research typically overlooks. Lastly, the study achieved complete insight into metabolic syndrome development pathways by including lifestyle variables like sleep duration, physical activity levels, and detailed nutritional intake alongside obesity and hypertension.

In conclusion, this study identified significant associations between hypertension, obesity, and key lifestyle behaviors among Korean working adults. Specifically, individuals with hypertension slept approximately 0.5 h less per weekday, and those with both obesity and hypertension reported significantly higher total energy intake and lower physical activity levels compared to their counterparts (all *p* < 0.01). These findings suggest that hypertension and obesity perpetuate a vicious cycle by contributing to reduced sleep duration, decreased physical activity, and increased energy intake, thereby exacerbating metabolic syndrome. Future studies should focus on establishing causal relationships using longitudinal data and objective measures such as accelerometers or biomarker-based dietary assessments. Given the interplay among these modifiable factors, it is also essential to translate these findings into practical interventions for the working population. Evidence-based interventions that promote healthy sleep, dietary habits, and physical activity should be prioritized. In particular, integrated health programs tailored for working adults—combining sleep hygiene education, nutritional counseling, and workplace-based physical activity initiatives—may offer a practical and impactful solution. Such programs should also account for occupational stress, long working hours, and irregular schedules common in this population.

## Data Availability

The original contributions presented in the study are included in the article/supplementary material, further inquiries can be directed to the corresponding author.

## References

[1] World Health Organization (2018) Obesity and overweight fact sheet. Geneva: World Health Organization.

[2] World Obesity Federation (2022) World Obesity Atlas 2022. World Obesity Federation. Available at: https://www.worldobesity.org/resources/resource-library/world-obesity-atlas-2022

[3] Albuquerque D Nóbrega C Manco L Padez C. The contribution of genetics and environment to obesity. Br Med Bull. (2017) 123:159–73. doi: 10.1093/bmb/ldx022, PMID: 28910990

[4] Ka S Choe YH Kim YI Kim N Seo M Choi Y. The effect of exercise interventions on sleep quality and weight loss in individuals with obesity: a systematic review and meta-analysis of randomized control trials. Appl Sci. (2025) 15:467. doi: 10.3390/app15010467 41120739

[5] Litwin M Kułaga Z. Obesity, metabolic syndrome, and primary hypertension. Pediatr Nephrol. (2021) 36:825–37. doi: 10.1007/s00467-020-04579-3, PMID: 32388582 PMC7910261

[6] Mills KT Stefanescu A He J. The global epidemiology of hypertension. Nat Rev Nephrol. (2020) 16:223–37. doi: 10.1038/s41581-019-0244-2, PMID: 32024986 PMC7998524

[7] Shariq OA McKenzie TJ. Obesity-related hypertension: a review of pathophysiology, management, and the role of metabolic surgery. Gland Surg. (2020) 9:80–93. doi: 10.21037/gs.2019.12.03, PMID: 32206601 PMC7082272

[8] Kjeldsen SE. Hypertension and cardiovascular risk: general aspects. Pharmacol Res. (2018) 129:95–9. doi: 10.1016/j.phrs.2017.11.003, PMID: 29127059

[9] Mathur P Jhawat V Shekhar S Dutt R Garg V Arora S. An overview of hypertension: pathophysiology, risk factors, and modern management. Curr Hypertens Rev. (2025). 21:64–81. doi: 10.2174/0115734021349254250224074832 40051353

[10] Tozo JVA Tadiotto MC Tozo TAA de Menezes-Junior FJ Mota J de Pereira BO. Effects of different physical exercise programs on blood pressure in overweight children and adolescents: systematic review and meta-analysis. BMC Pediatr. (2025) 25:252. doi: 10.1186/s12887-025-05575-y, PMID: 40155857 PMC11951679

[11] Antza C Kostopoulos G Mostafa S Nirantharakumar K Tahrani A. The links between sleep duration, obesity and type 2 diabetes mellitus. J Endocrinol. (2022) 252:125–41. doi: 10.1530/joe-21-0155, PMID: 34779405 PMC8679843

[12] Muscogiuri G Barrea L Annunziata G Di Somma C Laudisio D Colao A. Obesity and sleep disturbance: the chicken or the egg? Crit Rev Food Sci Nutr. (2019) 59:2158–65. doi: 10.1080/10408398.2018.1506979, PMID: 30335476

[13] Broussard JL Klein S. Insufficient sleep and obesity: cause or consequence. Obesity (Silver Spring). (2022) 30:1914–6. doi: 10.1002/oby.23539, PMID: 36042009 PMC9509457

[14] Suriyagandhi V Nachiappan V. Protective effects of melatonin against obesity-induced by leptin resistance. Behav Brain Res. (2022) 417:113598. doi: 10.1016/j.bbr.2021.113598, PMID: 34563600

[15] AlDabal L BaHammam AS. Metabolic, endocrine, and immune consequences of sleep deprivation. Open Respir Med J. (2011) 5:31–43. doi: 10.2174/1874306401105010031, PMID: 21754974 PMC3132857

[16] Lamon S Morabito A Arentson-Lantz E Knowles O Vincent GE Condo D. The effect of acute sleep deprivation on skeletal muscle protein synthesis and the hormonal environment. Phys Rep. (2021) 9:e14660. doi: 10.14814/phy2.14660, PMID: 33400856 PMC7785053

[17] Kim BH Lee HE. The association between working hours and sleep disturbances according to occupation and gender. Chronobiol Int. (2015) 32:1109–14. doi: 10.3109/07420528.2015.1064440, PMID: 26317888

[18] Kim KY Yun JM. Analysis of the association between health-related and work-related factors among workers and metabolic syndrome using data from the Korean National Health and nutrition examination survey (2016). Nutr Res Pract. (2019) 13:444–51. doi: 10.4162/nrp.2019.13.5.444, PMID: 31583064 PMC6760978

[19] Ryu H Chin DL. Factors associated with metabolic syndrome among Korean office workers. Arch Environ Occup Health. (2017) 72:249–57. doi: 10.1080/19338244.2016.1200004, PMID: 27285063

[20] Yun S Kim M Lee WT Yoon JH Won JU. Irregular work hours and the risk of sleep disturbance among Korean service workers required to suppress emotion. Int J Environ Res Public Health. (2021) 18:1517. doi: 10.3390/ijerph18041517, PMID: 33562866 PMC7915650

[21] Kim SY Kim SI Lim WJ. Association of sleep duration and working hours with suicidal ideation in shift workers: the Korean National Health and nutrition examination survey 2007–2018. Psychiatry Investig. (2021) 18:400–7. doi: 10.30773/pi.2020.0412, PMID: 33910326 PMC8169332

[22] Huh JH Lee JH Moon JS Sung KC Kim JY Kang DR. Metabolic syndrome severity score in Korean adults: analysis of the 2010–2015 Korea National Health and nutrition examination survey. J Korean Med Sci. (2019) 34:e48. doi: 10.3346/jkms.2019.34.e48, PMID: 30787681 PMC6374550

[23] Lee M Lee H Park J Kim HJ Kwon R Lee SW. Trends in hypertension prevalence, awareness, treatment, and control in South Korea, 1998–2021: a nationally representative serial study. Sci Rep. (2023) 13:21724. doi: 10.1038/s41598-023-49055-8, PMID: 38066091 PMC10709599

[24] Gwak DY Lee SA. Lifestyle behaviors according to the duration of hypertension: Korea National Health and nutrition examination survey 2016–2018. J Korean Med Sci. (2022) 37:e343. doi: 10.3346/jkms.2022.37.e343, PMID: 36536544 PMC9763709

[25] Yang HJ Kim MJ Hur HJ Lee BK Kim MS Park S. Association between Korean-style balanced diet and risk of abdominal obesity in Korean adults: an analysis using KNHANES-VI (2013–2016). Front Nutr. (2022) 8:772347. doi: 10.3389/fnut.2021.772347, PMID: 35127781 PMC8811126

[26] Yang HJ Park S Yoon TY Ryoo JH Park SK Jung JY. Nationwide changes in physical activity, nutrient intake, and obesity in South Korea during the COVID-19 pandemic era. Front Endocrinol. (2022) 13:965842. doi: 10.3389/fendo.2022.965842, PMID: 36176463 PMC9513223

[27] Bae YJ Kim MH Choi MK. Dietary mineral intake from nuts and its relationship to hypertension among Korean adults. Biol Trace Elem Res. (2022) 200:3519–28. doi: 10.1007/s12011-021-02952-3, PMID: 34661846

[28] Lee YJ Park YH Lee JW Sung ES Lee HS Park J. Household-specific physical activity levels and energy intakes according to the presence of metabolic syndrome in Korean young adults: Korean National Health and nutrition examination survey 2016–2018. BMC Public Health. (2022) 22:476–12. doi: 10.1186/s12889-022-12852-3, PMID: 35272663 PMC8908614

[29] Oh SW. Obesity and metabolic syndrome in Korea. Diabetes Metab J. (2011) 35:561–6. doi: 10.4093/dmj.2011.35.6.561, PMID: 22247896 PMC3253964

[30] Grundy SM Cleeman JI Bairey Merz CN Brewer HB Clark LT Hunninghake DB. Implications of recent clinical trials for the national cholesterol education program adult treatment panel III guidelines. J Am Coll Cardiol. (2004) 44:720–32. doi: 10.1016/j.jacc.2004.07.001, PMID: 15358046

[31] Grundy SM Brewer HB Jr Cleeman JI Smith SC Jr Lenfant C American Heart Association. Definition of metabolic syndrome: report of the National Heart, Lung, and Blood Institute/American Heart Association conference on scientific issues related to definition. Circulation. (2004) 109:433–8. doi: 10.1161/01.cir.0000111245.75752.c6, PMID: 14744958

[32] Seo MH Lee WY Kim SS Kang JH Kang JH Kim KK. 2018 Korean society for the study of obesity guideline for the management of obesity in Korea. J Obes Metab Syndr. (2019) 28:40–5. doi: 10.7570/jomes.2019.28.1.40, PMID: 31089578 PMC6484940

[33] Choi S-Y Han J-E Choi J Park M Sung S-H Sung AD-M. Association between sleep duration and symptoms of depression aged between 18 and 49: the Korea National Health and nutrition examination survey (KNHANES VII) from 2016 to 2018. Health. (2022) 10:2324. doi: 10.3390/healthcare10112324, PMID: 36421648 PMC9690060

[34] Armstrong T Bull F. Development of the world health organization global physical activity questionnaire (GPAQ). J Public Health. (2006) 14:66–70. doi: 10.1007/s10389-006-0024-x

[35] Hupin D Roche F Gremeaux V Chatard JC Oriol M Gaspoz JM. Even a low-dose of moderate-to-vigorous physical activity reduces mortality by 22% in adults aged≥ 60 years: a systematic review and meta-analysis. Br J Sports Med. (2015) 49:1262–7. doi: 10.1136/bjsports-2014-094306, PMID: 26238869

[36] Lee H Han E Kwon N j Kim Y Kim S Kim H. Korean Rural Development Administration's web based food and nutrient database management and validation system (NutriManager)–a report. J Food Compos Anal. (2017) 62:231–8. doi: 10.1016/j.jfca.2017.06.009

[37] Lee YH Kim SH. Prevalence of obesity, its comorbidities, and related risk factors among young Korean adults: using the Korea National Health and nutrition examination survey (2019–2021). Korean J Fam Pract. (2024) 14:211–9. doi: 10.21215/kjfp.2024.14.4.211

[38] Bozkurt B Aguilar D Deswal A Dunbar SB Francis GS Horwich T. Contributory risk and management of comorbidities of hypertension, obesity, diabetes mellitus, hyperlipidemia, and metabolic syndrome in chronic heart failure: a scientific statement from the American Heart Association. Circulation. (2016) 134:e535–78. doi: 10.1161/cir.0000000000000450, PMID: 27799274

[39] Fujita K Nishizawa H Funahashi T Shimomura I Shimabukuro M. Systemic oxidative stress is associated with visceral fat accumulation and the metabolic syndrome. Circ J. (2006) 70:1437–42. doi: 10.1253/circj.70.1437, PMID: 17062967

[40] Ziolkowska S Binienda A Jabłkowski M Szemraj J Czarny P. The interplay between insulin resistance, inflammation, oxidative stress, base excision repair and metabolic syndrome in nonalcoholic fatty liver disease. Int J Mol Sci. (2021) 22:11128. doi: 10.3390/ijms222011128, PMID: 34681787 PMC8537238

[41] Hall ME do Carmo JM da Silva AA Juncos LA Wang Z Hall JE. Obesity, hypertension, and chronic kidney disease. Int J Nephrol Renov Dis. (2014) 7:75–88. doi: 10.2147/ijnrd.s39739, PMID: 24600241 PMC3933708

[42] Ferroni P Basili S Paoletti V Davi G. Endothelial dysfunction and oxidative stress in arterial hypertension. Nutrition, Metabolism and Cardiovascular Diseases. (2006) 16:222–233. doi: 10.1016/j.numecd.2005.11.012 16580590

[43] Incalza MA D’Oria R Natalicchio A Perrini S Laviola L Giorgino F. Oxidative stress and reactive oxygen species in endothelial dysfunction associated with cardiovascular and metabolic diseases. Vascular Pharmacology. (2018) 100:1–19. doi: 10.1016/j.vph.2017.05.005 28579545

[44] Redon J Cifkova R Laurent S Nilsson P Narkiewicz K Erdine S. Mechanisms of hypertension in the cardiometabolic syndrome. J Hypertens. (2009) 27:441–51. doi: 10.1097/hjh.0b013e32831e13e5, PMID: 19262221

[45] Stanciu S Rusu E Miricescu D Radu AC Axinia B Vrabie AM. Links between metabolic syndrome and hypertension: the relationship with the current antidiabetic drugs. Meta. (2023) 13:87. doi: 10.3390/metabo13010087, PMID: 36677012 PMC9863091

[46] Chong MY Han I. Distribution of the metabolic syndrome by obesity and health behavior based on the eighth KNHANES at 2019. J Korean Soc Food Sci Nutr. (2022) 51:1136–47. doi: 10.3746/jkfn.2022.51.11.1136

[47] Yang HI Kim HC Jeon JY. The association of resting heart rate with diabetes, hypertension, and metabolic syndrome in the Korean adult population: the fifth Korea National Health and nutrition examination survey. Clin Chim Acta. (2016) 455:195–200. doi: 10.1016/j.cca.2016.01.006, PMID: 26778411

[48] Choi JE Lee HA Park SW Lee JW Lee JH Park H. Increase of prevalence of obesity and metabolic syndrome in children and adolescents in Korea during the COVID-19 pandemic: a cross-sectional study using the KNHANES. Children (Basel). (2023) 10:1105. doi: 10.3390/children10071105, PMID: 37508602 PMC10378374

[49] Guo X Zheng L Wang J Zhang X Zhang X Li J. Epidemiological evidence for the link between sleep duration and high blood pressure: a systematic review and meta-analysis. Sleep Med. (2013) 14:324–32. doi: 10.1016/j.sleep.2012.12.001, PMID: 23394772

[50] Chaput JP Després JP Bouchard C Tremblay A. The association between sleep duration and weight gain in adults: a 6-year prospective study from the Quebec family study. Sleep. (2008) 31:517–23. doi: 10.1093/sleep/31.4.517, PMID: 18457239 PMC2279744

[51] Baek SU Won JU Yoon JH. Gender differences in the association between long work hours, weekend work, and insomnia symptoms in a nationally representative sample of workers in Korea. Sleep Health. (2025) 11:191–7. doi: 10.1016/j.sleh.2024.11.002, PMID: 39757055

[52] Son SM Park EJ Cho YH Lee SY Choi JI Lee YI. Association between weekend catch-up sleep and metabolic syndrome with sleep restriction in Korean adults: a cross-sectional study using KNHANES. Diabetes Metab Syndr Obes. (2020) 13:1465–71. doi: 10.2147/dmso.s247898, PMID: 32431530 PMC7200717

[53] Cho KH Cho EH Hur J Shin D. Association of sleep duration and obesity according to gender and age in Korean adults: results from the Korea national health and nutrition examination survey 2007–2015. J Korean Med Sci. (2018) 33:e345. doi: 10.3346/jkms.2018.33.e345, PMID: 30595686 PMC6306326

[54] Park SE Kim HM Kim DH Kim J Cha BS Kim DJ. The association between sleep duration and general and abdominal obesity in Koreans: data from the Korean National Health and nutrition examination survey, 2001 and 2005. Obesity (Silver Spring). (2009) 17:767–71. doi: 10.1038/oby.2008.586, PMID: 19180067

[55] Hall KD Sacks G Chandramohan D Chow CC Wang YC Gortmaker SL. Quantification of the effect of energy imbalance on bodyweight. Lancet. (2011) 378:826–37. doi: 10.1016/s0140-6736(11)60812-x, PMID: 21872751 PMC3880593

[56] Togo P Osler M Sørensen T Heitmann B. Food intake patterns and body mass index in observational studies. Int J Obes Relat Metab Disord. (2001) 25:1741–51. doi: 10.1038/sj.ijo.0801819, PMID: 11781753

[57] Ha K Kim K Chun OK Joung H Song Y. Differential association of dietary carbohydrate intake with metabolic syndrome in the US and Korean adults: data from the 2007–2012 NHANES and KNHANES. Eur J Clin Nutr. (2018) 72:848–60. doi: 10.1038/s41430-017-0031-8, PMID: 29339830

[58] Shin S Kim SA Ha J Lim K. Sugar-sweetened beverage consumption in relation to obesity and metabolic syndrome among Korean adults: a cross-sectional study from the 2012–2016 Korean National Health and nutrition examination survey (KNHANES). Nutrients. (2018) 10:1467. doi: 10.3390/nu10101467, PMID: 30304842 PMC6213560

[59] Subar AF Freedman LS Tooze JA Kirkpatrick SI Boushey C Neuhouser ML. Addressing current criticism regarding the value of self-report dietary data. J Nutr. (2015) 145:2639–45. doi: 10.3945/jn.115.219634, PMID: 26468491 PMC4656907

[60] Lee K Hong KS Park J Park W. Readjustment of circadian clocks by exercise intervention is a potential therapeutic target for sleep disorders: a narrative review. Phys Act Nutr. (2024) 28:35–42. doi: 10.20463/pan.2024.0014, PMID: 39097996 PMC11298283

[61] Lee Y Kim N Go S Kim J Park J. Sugary snack restriction enhances body composition improvement in overweight women engaging in non-face-to-face walking during COVID-19. Front Public Health. (2024) 12:1396598. doi: 10.3389/fpubh.2024.1396598, PMID: 38887258 PMC11180888

[62] Qin P Li Q Zhao Y Chen Q Sun X Liu Y. Sugar and artificially sweetened beverages and risk of obesity, type 2 diabetes mellitus, hypertension, and all-cause mortality: a dose–response meta-analysis of prospective cohort studies. Eur J Epidemiol. (2020) 35:655–71. doi: 10.1007/s10654-020-00655-y, PMID: 32529512

[63] Siervo M Montagnese C Mathers JC Soroka KR Stephan BC Wells JC. Sugar consumption and global prevalence of obesity and hypertension: an ecological analysis. Public Health Nutr. (2014) 17:587–96. doi: 10.1017/s1368980013000141, PMID: 23414749 PMC10282320

[64] Donnelly JE Hill JO Jacobsen DJ Potteiger J Sullivan DK Johnson SL. Effects of a 16-month randomized controlled exercise trial on body weight and composition in young, overweight men and women: the Midwest exercise trial. Arch Intern Med. (2003) 163:1343–50. doi: 10.1001/archinte.163.11.1343, PMID: 12796071

[65] Diaz KM Shimbo D. Physical activity and the prevention of hypertension. Curr Hypertens Rep. (2013) 15:659–68. doi: 10.1007/s11906-013-0386-8, PMID: 24052212 PMC3901083

[66] Hegde SM Solomon SD. Influence of physical activity on hypertension and cardiac structure and function. Curr Hypertens Rep. (2015) 17:77–8. doi: 10.1007/s11906-015-0588-3, PMID: 26277725 PMC4624627

[67] Esposito K Chiodini P Colao A Lenzi A Giugliano D. Metabolic syndrome and risk of cancer: a systematic review and meta-analysis. Diabetes Care. (2012) 35:2402–11. doi: 10.2337/dc12-0336, PMID: 23093685 PMC3476894

[68] Ryu M Lee S Gym H Baek WC Kimm H. Analysis of association of occupational physical activity, leisure-time physical activity, and sedentary lifestyle with hypertension according to the adherence with aerobic activity in women using Korea National Health and nutrition examination survey 2016-2017 data. Int J Hypertens. (2020) 2020:8943492. doi: 10.1155/2020/8943492, PMID: 32110448 PMC7042501

[69] Prince SA Adamo KB Hamel M Hardt J Connor Gorber S Tremblay M. A comparison of direct versus self-report measures for assessing physical activity in adults: a systematic review. Int J Behav Nutr Phys Act. (2008) 5:56. doi: 10.1186/1479-5868-5-56, PMID: 18990237 PMC2588639

[70] Kim N Ka S Park J. Effects of exercise timing and intensity on physiological circadian rhythm and sleep quality: a systematic review. Phys Act Nutr. (2023) 27:052–63. doi: 10.20463/pan.2023.0029, PMID: 37946447 PMC10636512

